# Effects of a deep learning-based image quality enhancement method on a digital-BGO PET/CT system for ^18^F-FDG whole-body examination

**DOI:** 10.1186/s40658-025-00742-7

**Published:** 2025-03-28

**Authors:** Kenta Miwa, Shin Yamagishi, Shun Kamitaki, Kouichi Anraku, Shun Sato, Tensho Yamao, Noriaki Miyaji, Kaito Wachi, Naochika Akiya, Kei Wagatsuma, Kazuhiro Oguchi

**Affiliations:** 1 Department of Radiological Sciences, School of Health Sciences, Fukushima Medical University, 10-6 Sakaemachi, Fukushima-Shi, Fukushima, 960-8516 Japan; 2 Center of Radiology and Diagnostic Imaging, Aizawa Hospital, 2-5-1 Honjo, Matsumoto-Shi, Nagano, 390-8510 Japan; 3 School of Allied Health Sciences, Kitasato University, 1-15-1 Kitazato, Minami-Ku, Sagamihara, Kanagawa 252-0373 Japan

**Keywords:** PET/CT, Deep-learning, BSREM

## Abstract

**Background:**

The digital-BGO PET/CT system, Omni Legend 32, incorporates modified block sequential regularized expectation maximization (BSREM) image reconstruction and a deep learning-based time-of-flight (TOF)-like image quality enhancement process called Precision DL (PDL). The present study aimed to define the fundamental characteristics of PDL using phantom and clinical images.

**Methods:**

A NEMA IEC body phantom was scanned using the Omni Legend 32 PET/CT system. All PET/CT images were acquired over 60 and 90 s per bed position, with a 384 × 384 matrix. Phantom images were reconstructed using OSEM + PSF and BSREM at β values of 100–1,000, combined with low (LPDL), medium (MPDL), and high (HPDL) PDL. We evaluated contrast recovery, background variability, and the contrast-to-noise ratio (CNR) of a 10 mm hot sphere. Thirty clinical whole-body ^18^F-FDG PET/CT examinations were included. Clinical images were reconstructed using OSEM + PSF and BSREM at β values of 200, 300, 400, 500, and 600, determined based on findings from the phantom study, combined with the three PDL models. Noise levels, mean SUV (SUV_mean_), and the signal-to-noise ratio (SNR) of the liver as well as signal-to-background ratios (SBR) and maximum SUV (SUV_max_) of lesions were evaluated. Two blinded readers evaluated visual image quality and rated several aspects to complement the analysis.

**Results:**

Contrast recovery and background variability decreased as the β value increased. This trend was consistent even when PDL processing was added to BSREM. Increased strength of the PDL models led to higher CNR. Noise levels decreased as a function of increasing β values in BSREM, resulting in a higher SNR, but lower SBR. Combining PDL with BSREM resulted in all β values producing better results in terms of noise, SBR, and SNR than OSEM + PSF. As the PDL increased (LPDL < MPDL < HPDL), noise levels, SBR, and SNR became higher. The β values of 400, 200, 300, and 300 for BSREM, LPDL, MPDL, and HPDL, respectively, resulted in noise equivalent to OSEM + PSF but significantly increased the SUV_max_ (9%, 15%, 18%, and 27%), SBR (16%, 17%, 20%, and 32%), and SNR (17%, 19%, 31%, and 36%), respectively. The visual evaluation of image quality yielded similar scores across BSREM + PDL reconstructions, although BSREM with β = 600 combined with MPDL delivered the best overall image quality and total mean score.

**Conclusion:**

The combination of BSREM and PDL significantly enhanced the SUV_max_ of lesions and image quality compared with OSEM + PSF. A combination of BSREM at β values of 500–600 and MPDL is recommended for oncological whole-body PET/CT imaging when using PDL on the Omni Legend.

## Introduction

Positron emission tomography/computed tomography (PET/CT) with ^18^F-fluoro-2-deoxy-D-glucose (^18^F-FDG) is widely used to stage malignancies and assess treatment responses [1]. However, the spatial resolution of PET is relatively low, which can hamper the detection of small lesions due to the partial volume effect (PVE) [2]. Semiquantitative evaluation with ^18^F-FDG PET to differentiate benign from malignant lesions such as pulmonary nodules and mediastinal lymph nodes in patients with lung cancer, is challenging because standardized uptake values (SUVs) can be unreliable [3, 4]. This is partly because technical factors such as scanner type, image reconstruction methods, and image processing techniques can affect the accuracy and reproducibility of the SUV [5–7].

An iterative Bayesian penalized likelihood (BPL) algorithm has been incorporated into the commercial software Q.Clear (GE HealthCare, Milwaukee, WI, USA), that is implemented in GE HealthCare PET/CT systems [8]. This algorithm achieves accurate image reconstruction using a penalty function to reduce noise and by incorporating point spread function (PSF) modeling [9]. The modified block sequential regularized expectation maximization (BSREM) serves as a numerical optimizer to maximize the BPL penalty function for Q.Clear [10]. Compared with conventional ordered subset expectation maximization (OSEM) reconstruction, BSREM significantly improves spatial resolution, image quality and the SUV of small lesions [2, 11, 12]. Q.Clear software requires only one user input variable, the penalization factor (β), which controls overall noise suppression [13]. Several studies have focused on optimizing the β value in BSREM [14–17].

The Omni Legend 32 PET/CT system (Omni Legend; GE HealthCare) features a new digital detection technology that combines bismuth germanium oxide (BGO) crystals with a silicon photomultiplier (SiPM) and extends the axial field of view to 32 cm [18, 19]. We recently found that the Omni Legend delivers class-leading sensitivity and count rates while maintaining high spatial resolution comparable to other current SiPM-based PET systems, thus substantially improving image quality and enhancing quantitation metrics such as the SUV [18].

The new deep-learning, image processing algorithm Precision DL (PDL; GE HealthCare) was developed to overcome the limitations of the BGO PET/CT system, which cannot provide time-of-flight (TOF) information [18]. Precision DL leverages deep learning to simulate the effects of TOF in PET images that are reconstructed without TOF data [20]. This represents the first attempt to convert non-TOF PET images into TOF-like images. Unlike previous deep learning methods that have primarily focused on reducing noise on PET images [21–23], PDL is fundamentally different because it addresses the complex ways in which TOF information affects image properties [24, 25]. Instead of adding TOF data to PET coincidence events, neural networks are trained to learn how TOF information alters various aspects of PET image properties and replicates them when processing images with non-TOF input. Specifically, to transform non-TOF images reconstructed by BSREM with a range of β values into TOF BSREM images with specific regularization settings and desired contrast-to-noise ratios, low (LPDL), medium (MPDL), and high (HPDL) models were trained in a supervised learning session [20]. Each model offers a different balance between contrast enhancement and noise suppression. PDL is specifically designed to work exclusively with BSREM for image reconstruction. After data acquisition on an Omni Legend scanner, users can select a β value for BSREM reconstruction and specify the strength of PDL (LPDL, MPDL, or HPDL). The implementation of PDL processing by developers has been discussed [20, 24, 25], but the specific characteristics of PDL as implemented in the Omni Legend system have not been investigated as far as we can ascertain. The present study aimed to define the fundamental characteristics of PDL using phantom and clinical images, and to determine relationships among β values, BSREM and the PDL models.

## Materials and methods

### Data acquisition

*PET System.* We acquired PET images using the Omni Legend with an integrated SiPM-based detector combined with BGO scintillator crystals (digital BGO). The PET scanner is equipped with a detector consisting of 22 modules, each containing 24 BGO blocks for a total of 528. An array of 6,312 crystals (4.1 × 4.1 × 30 mm each) within each block are paired with 18 SiPMs (6 × 6 mm each) arranged in a 3 × 6 configuration [18]. This results in a total of 38,016 BGO crystals and 9,504 SiPM channels that cover axial and transaxial fields of view (FOV) of 32 and 70 cm, respectively. The sensitivity of the scanner is 47.30 cps/kBq, and the spatial resolution of OSEM is 3.7 mm at 1 cm from the center of the FOV. The scatter fraction, count rate accuracy, and peak noise-equivalent count rates were 35.4%, 1.7%, and 501.7 kcps, respectively, at 15.7 kBq/mL [18].

*Phantom.* We used a National Electrical Manufacturers Association International Electrotechnical Commission (NEMA IEC) body phantom containing spheres with ⌀ 10–37 (NEMA IEC Body Phantom Set™; Data Spectrum Corp., Durham, NC, USA) [26, 27]. The target-to-background ratio (TBR) in the phantom was 4:1 on a background activity concentration of 2.53 kBq/mL [6].

*Clinical Data.* The Ethics Committee at Aizawa Hospital approved this study (Approval No. 2023–020), which proceeded according to the ethical principles enshrined in the Declaration of Helsinki (2013 amendment). We included 30 patients (20 men and 10 women; mean age, 75 ± 8 years; age range, 53–87 years) who were clinically assessed by whole-body ^18^F-FDG PET/CT imaging using the Omni Legend between January 2023 and May 2023. The first 30 patients with positive pulmonary lesions observed during this period were selected. Of these patients, 23 underwent imaging to evaluate pulmonary nodules, 3 for staging non-small cell lung cancer, and 4 for staging colorectal cancer with suspected metastases. We acquired PET/CT images at 60 min after injecting each patient with 3.7 ± 0.1 MBq/kg (mean ± SD; range, 3.3–3.9 MBq/kg) ^18^F-FDG. The patients were scanned in four bed positions for 90 s each.

### Image reconstruction

*Phantom.* Images acquired for 60 (re-binned from list-mode data) and 90 s/bed position were reconstructed using OSEM + PSF (iterations, 4; subsets, 12; Gaussian postfilter, 2 mm) [28, 29] and BSREM combined with LPDL, MPDL, and HPDL models. The β value varied from 100 to 1,000 at intervals of 100 in BSREM. The FOV was 50 cm, with a matrix of 384 × 384 and a slice thickness of 2.08 mm per slice.

*Clinical Data.* Images acquired for 60 and 90 s/bed were reconstructed according to the following parameters: OSEM + PSF (iteration, 4; subsets, 12; 2-mm Gaussian postfilter) and BSREM at β values of 200, 300, 400, 500, and 600 (based on the phantom findings), LPDL, MPDL, and HPDL, motion free switched on [30], a 384 × 384 matrix and a 60-cm FOV.

### Image analysis

*Phantom.* We evaluated contrast recovery, background variability, and the contrast-to-noise ratio (CNR) of the 10 mm hot sphere to assess image quality [6]. A circular region of interest (ROI) was placed on the sphere at the center of its axial slice. We also placed twelve 10-mm circular ROIs in the background area, on the central slice and on slices 1 and 2 cm above and below it, resulting in a total of 60 ROIs. We calculated contrast recovery of the 10 mm hot sphere as:

$$Contrast\,recovery \left( \% \right) = \frac{{\frac{{C_{H} }}{{C_{B} }} - 1}}{{\frac{{a_{H} }}{{a_{B} }} - 1}} \times 100,$$

where $C_{H}$ and $C_{B}$ represent the average activity in the hot spheres and background ROIs, respectively, and $\frac{{a_{H} }}{{a_{B} }}$ is the activity concentration ratio between the hot spheres and the background. Background variability (%), a measure of image noise, was defined as the standard deviation (SD) of mean activity across the 60 background ROIs divided by their mean activity. The CNR was then determined by dividing contrast recovery by background variability. All data were processed and analyzed using PMOD v.4.4 (Bruker, Billerica, MA, USA).

*Clinical Data.* We evaluated noise, mean SUV (SUV_mean_) and the signal-to-noise ratio (SNR) of the liver as well as the signal-to-background ratio (SBR) and maximum SUV (SUV_max_) of lesions in clinical PET images [11]. Liver noise was defined as the SD normalized to the SUV_mean_ of an entire-liver volume of interest (VOI), which was established by segmenting the liver from CT images of each patient. We calculated SNR by dividing the lesion SUV_max_ by liver noise, and determined SBR was by dividing the lesion SUV_max_ by the SUV_mean_ of the entire-liver reference. We created VOIs of lesions on PET images using region-growing and thresholding (41% of the maximum voxel value) [11, 31]. All data were processed and analyzed using Xeleris V (Q.Volumetrix AI; GE HealthCare).

*Visual evaluation.* Two certified nuclear medicine technologists, each with 17 and 12 years of experience in nuclear medicine independently visually assessed image quality. Five PET/CT assessments were evaluated, each comprising 21 reconstructions (OSEM and BSREM at β values of 200, 300, 400, 500, and 600, with and without LPDL, MPD, or HPDL models). All data were rendered innominate and the readers were blinded to the reconstruction methods. The images were visualized using an Xeleris workstation. The PET image sets were randomly reviewed individually and the parameters of overall image quality, contrast, sharpness, artifacts, noise level, liver background homogeneity, and lesion detectability were rated as poor (1), moderate (2), good (3), and very good (4) [11].

*Statistical analysis.* Inter-reader agreement between the two readers was determined using intraclass correlation coefficients (ICCs), which are commonly used to assess the consistency of visual evaluations. Differences among the different reconstruction methods were assessed using Friedman tests, which were chosen as a non-parametric alternative to repeated measures ANOVA to compare multiple related groups, specifically the reconstruction methods applied to the same dataset. When the Friedman tests indicated significant differences, Wilcoxon signed-rank tests with Bonferroni correction were performed to determine which specific pairs of reconstruction conditions differed. Relationships between visually evaluated parameters were assessed using Spearman rank correlation coefficients, as this method does not require the data to follow a normal distribution and is effective for evaluating the strength and direction of relationships between ranked variables. All data were statistically analyzed using IBM SPSS Statistics for Windows, Version 29.0.2.029 (IBM Corp., Armonk, NY, USA) and values with *p* < 0.05 were considered statistically significant.

## Results

### NEMA phantom

**Fig. 1:**
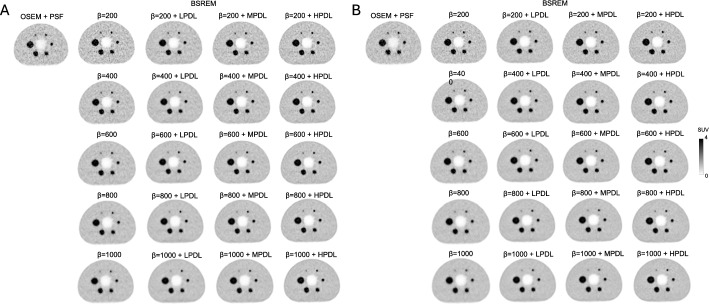
Representative axial images of central slice in NEMA body phantom acquired at 60 (**A**) and 90 s/bed (**B**) and reconstructed using PDL models

Figure 1 shows representative axial images of the central slice acquired for 60- and 90-s/bed in the NEMA body phantom reconstructed using the PDL models. The PVE tended to be less obvious, whereas the background noise became more apparent as strength increased from LPDL to HPDL. The PET image for 90-s/bed reconstructed using BSREM was visually comparable to that of PET at 60 s/bed when BSREM was combined with MPDL and HPDL. In addition, an under-estimated artifact was observed near the largest spheres (22 mm and 37 mm) on several reconstructed phantom images in both 60- and 90-s/bed acquisitions, particularly with HPDL.

**Fig. 2:**
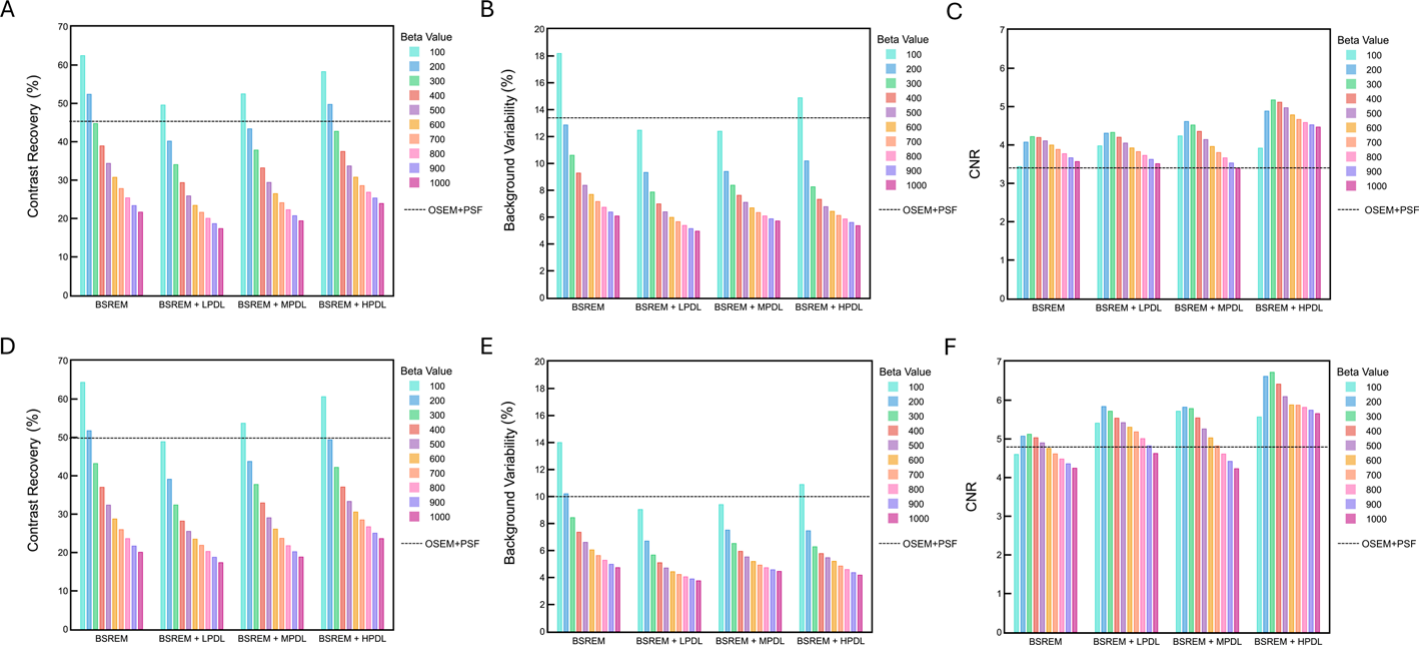
Contrast recovery (**A**, **D**), background variability (**B**, **E**), and CNR (**C**, **F**) of 10-mm spheres as a function of β for PDL models. Images were acquired for 60 (A–C) and 90 (D–F) s/bed. CNR, contrast-noise ratio; HPDL, high-precision DL; LPDL, low-precision DL; MPDL, medium-precision DL

Figure 2 shows that CNR was consistently higher using BSREM (β = 200‒600), than OSEM, regardless of PDL. Contrast recovery and background variability consistently decreased as the β value increased even when PDL processing was added to BSREM. Contrast recovery improved as the strength of the PDL models increased, which in turn led to a higher CNR. Except for β = 100, the combination of BSREM and HPDL resulted in the highest CNR among all β values. The CNR was highest for BSREM + LPDL and MPDL at β = 200, and for BSREM + HPDL, at β = 300. The CNR provided by this combination acquired at 60-s/bed was comparable to that of 90-s/bed PET images reconstructed using BSREM.

### Clinical data

**Fig. 3:**
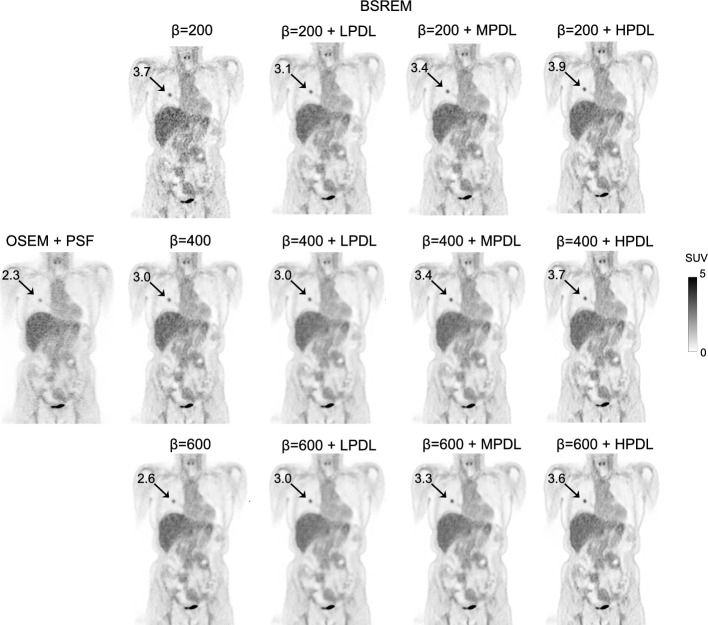
Representative coronal PET images of non-small cell lung cancer acquired using Omni Legend at 90-s/bed acquisition. Images were reconstructed using OSEM + PSF (iterations, 4; subsets, 12; 2-mm Gaussian postfilter) and BSREM at β values of 200, 400, and 600, with or without LPDL, MPDL, or HPDL strength. Numbers indicate lesion SUV_max_. HPDL, high-precision DL; LPDL, low-precision DL; MPDL, medium-precision DL; PSF, point spread function

We analyzed 30 clinical PET/CT images of 45 pulmonary lesions measuring 4.4 ± 6.8 (0.3–29.0) cm^3^, with SUV_max_ 6.6 ± 5.8 (0.4–27.2) determined from OSEM images. Table 1 shows the SUV_max_ of lesions measured in 90-s/bed images reconstructed using BSREM. Table 2 shows that the mean liver reference volume derived from the CT images was 1,014.8 ± 227.4 (725.4–1,614.0) cm^3^ with SUV_mean_ 2.2 ± 0.2 (1.9–3.0). Figure 3 shows representative PET images of how the choice of regularization value and the strength of the PDL model visually influenced image quality and quantitation.

**Table 1:** Quantitative measures of the lesions using OSEM and BSREM combined with PDL

			Measure	
Reconstruction			SUV_max_	SUV_mean_
OSEM PSF			6.6 (0.4–27.2)	3.1 (0.4–8.9)
BSREM	β = 200	Non-PDL	8.2 (0.8–27.6)	3.8 (0.6–10.4)
		LPDL	7.6 (0.5–26.2)	3.6 (0.4–10.1)
		MPDL	8.0 (0.6–26.9)	3.7 (0.4–10.5)
		HPDL	8.6 (0.5–26.6)	3.7 (0.4–10.8)
	β = 300	Non-PDL	7.6 (0.6–25.9)	3.6 (0.5–10.2)
		LPDL	7.2 (0.4–24.8)	3.5 (0.4–10.1)
		MPDL	7.8 (0.5–25.6)	3.7 (0.4–10.4)
		HPDL	8.4 (0.5–25.4)	3.8 (0.4–10.7)
	β = 400	Non-PDL	7.2 (0.5–24.9)	3.5 (0.4–10.1)
		LPDL	7.1 (0.4–24.0)	3.5 (0.4–10.1)
		MPDL	7.7 (0.5–24.8)	3.7 (0.4–10.5)
		HPDL	8.3 (0.5–24.7)	3.8 (0.4–10.6)
	β = 500	Non-PDL	6.9 (0.5–24.2)	3.5 (0.4–9.9)
		LPDL	7.0 (0.4–23.4)	3.5 (0.3–10.1)
		MPDL	7.6 (0.4–24.3)	3.7 (0.4–10.5)
		HPDL	8.2 (0.5–24.2)	3.8 (0.4–10.6)
	β = 600	Non-PDL	6.7 (0.4–23.6)	3.4 (0.4–9.8)
		LPDL	6.9 (0.4–23.0)	3.4 (0.3–10.0)
		MPDL	7.5 (0.4–23.9)	3.6 (0.4–10.4)
		HPDL	8.1 (0.5–23.7)	3.8 (0.4–10.6)

Data are presented as mean followed by range. OSEM was performed with 4 iterations, 12 subsets, 2-mm gaussian postprocessing filter, and PSF. BSREM was combined with PDL with β value of 200, 300, 400, 500, 600; scan duration was 90 s/bed.

**Table 2:** Quantitative measures of the entire-liver reference using OSEM and BSREM combined with PDL

			Measure			
Reconstruction			SUV_max_	SUV_mean_	SUV_SD_	Noise level
OSEM PSF			4.2 (3.0–7.6)	2.2 (1.9–3.0)	0.4 (0.3–0.6)	0.18 (0.14–0.23)
BSREM	β = 200	Non-PDL	5.3 (4.0–9.2)	2.3 (2.0–3.1)	0.5 (0.4–0.6)	0.21 (0.15–0.27)
		LPDL	4.4 (3.1–8.6)	2.3 (1.9–3.1)	0.4 (0.3–0.6)	0.18 (0.13–0.24)
		MPDL	4.6 (3.2–9.3)	2.3 (1.9–3.1)	0.4 (0.3–0.6)	0.18 (0.13–0.24)
		HPDL	4.8 (3.4–9.7)	2.3 (2.0–3.1)	0.4 (0.3–0.6)	0.19 (0.14–0.25)
	β = 300	Non-PDL	4.6 (3.1–8.9)	2.3 (2.0–3.1)	0.4 (0.3–0.6)	0.19 (0.14–0.25)
		LPDL	4.1 (2.8–8.4)	2.3 (1.9–3.0)	0.4 (0.3–0.5)	0.17 (0.12–0.23)
		MPDL	4.3 (2.9–9.4)	2.3 (1.9–3.1)	0.4 (0.3–0.6)	0.17 (0.13–0.24)
		HPDL	4.5 (3.1–9.7)	2.3 (2.0–3.1)	0.4 (0.3–0.6)	0.18 (0.14–0.25)
	β = 400	Non-PDL	4.4 (3.1–9.2)	2.3 (2.0–3.1)	0.4 (0.3–0.6)	0.18 (0.13–0.24)
		LPDL	4.0 (2.7–8.7)	2.3 (1.9–3.0)	0.3 (0.3–0.5)	0.16 (0.12–0.23)
		MPDL	4.2 (2.8–9.7)	2.3 (1.9–3.1)	0.4 (0.3–0.6)	0.17 (0.12–0.24)
		HPDL	4.5 (3.0–9.7)	2.3 (2.0–3.1)	0.4 (0.3–0.6)	0.18 (0.13–0.25)
	β = 500	Non-PDL	4.2 (3.0–9.0)	2.3 (1.9–3.1)	0.4 (0.3–0.6)	0.17 (0.13–0.24)
		LPDL	4.0 (2.6–8.5)	2.3 (1.9–3.0)	0.4 (0.2–0.5)	0.16 (0.11–0.23)
		MPDL	4.2 (2.8–9.6)	2.3 (1.9–3.1)	0.4 (0.3–0.5)	0.17 (0.12–0.24)
		HPDL	4.4 (3.0–10.0)	2.3 (2.0–3.1)	0.4 (0.3–0.6)	0.17 (0.13–0.25)
	β = 600	Non-PDL	4.0 (2.8–7.8)	2.3 (1.9–3.1)	0.4 (0.3–0.6)	0.17 (0.12–0.23)
		LPDL	3.9 (2.6–8.1)	2.3 (1.9–3.0)	0.4 (0.2–0.5)	0.16 (0.11–0.22)
		MPDL	4.1 (2.7–9.0)	2.3 (1.9–3.1)	0.4 (0.3–0.5)	0.17 (0.12–0.24)
		HPDL	4.3 (2.9–9.8)	2.3 (2.0–3.1)	0.4 (0.3–0.6)	0.17 (0.13–0.25)

Data are presented as mean followed by range. OSEM was performed with 4 iterations, 12 subsets, 2-mm gaussian postprocessing filter, and PSF. BSREM was combined with PDL with β value of 200, 300, 400, 500, 600; scan duration was 90 s/bed.

**Fig. 4:**
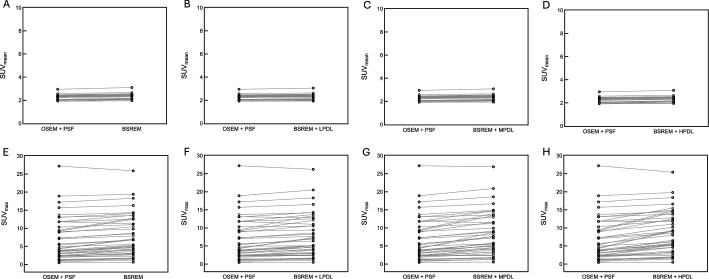
Clinical comparison of liver SUV_mean_ (**A**–**D**) and lesion SUV_max_ (**E**–**G**) using PDL models from 90-s/bed PET images under optimal conditions determined by phantom evaluation (BSREM with LPDL/MPDL at β = 200 and BSREM with HPDL at β = 300). HPDL, high-precision DL; LPDL, low-precision DL; MPDL, medium-precision DL

Figure 4 shows that the liver SUV_mean_ did not significantly differ between the reconstruction methods, but lesion SUV_max_ was higher for images reconstructed using BSREM than OSEM, and was further increased with PDL.

**Fig. 5:**
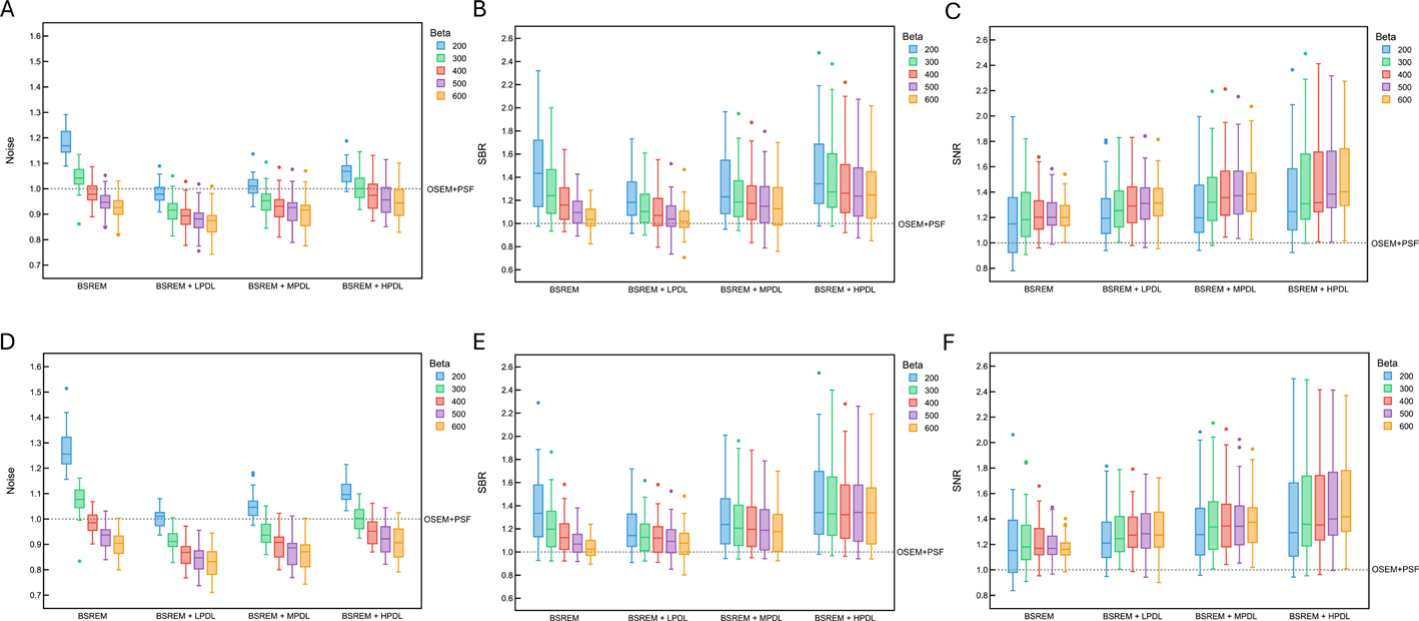
Noise (**A**, **D**), SBR (**B**, **E**), and SNR (**C**, **F**) on clinical 60 and 90-s/bed PET images as a function of β value and PDL models compared with OSEM. (A–C) 60- and (D–F) and 90-s/bed acquisition images. HPDL, high-precision DL; LPDL, low-precision DL; MPDL, medium-precision DL; noise, liver noise; SBR, signal-to-background ratio; SNR, lesion signal-to-noise ratio. Dashed line represents OSEM results

Figure 5 shows that noise levels tended to be lower and SBR and SNR were higher in images reconstructed with BSREM than OSEM, regardless of PDL. Noise levels decreased as a function of increasing β in BSREM, resulting in a higher SNR and lower SBR. All β values produced better noise level, SBR, and SNR when BSREM was accompanied with, than without PDL. Noise levels and SBR increased as the strength of PDL increased. The SNRs for 60- and 90-s/bed image acquisition were clearly improved by BSREM + HPDL, although the effects of HPDL on SNR varied among patients.

**Fig. 6:**
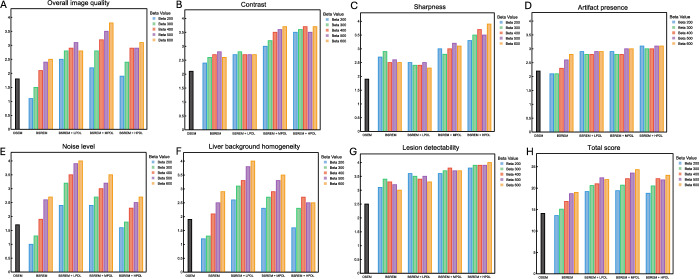
Visually scored image quality. Two readers applied an arbitrary 4-point scale (1, poor; 2, moderate; 3, good; 4, very good) to assess different aspects (**A**–**G**) of image quality. (**H**) Total mean scores. Each of five patients had 21 sets of images that were reconstructed using OSEM + PSF (4 iterations, 12 subsets, 2-mm Gaussian postfilter) and BSREM at β values of 200, 400, and 600, with and without LPDL, MPDL, or HPDL and 90-s/bed image acquisition. HPDL, high-precision DL; LPDL, low-precision DL; MPDL, medium-precision DL

The ICC values of 0.92, 0.89, 0.86, 0.83, 0.71, 0.85, and 0.81, respectively (all *p* < 0.05), indicated excellent inter-reader agreement over overall image quality, contrast, sharpness, artifacts, noise, liver background homogeneity, and lesion detectability. Figure 6 shows that reconstruction using BSREM combined with β = 600 and MPDL was ranked as optimal in terms overall visual image quality and the highest total mean score across all aspects. Both OSEM and BSREM at all β values significantly differed from BSREM + MPDL at β values of 500 and 600, and BSREM + HPDL at β values of 400, 500, and 600 (*p* < 0.05). The β values within each PDL model did not significantly differ. The individual parameters, contrast, sharpness, and lesion detectability tended to correlate (*p* < 0.05) in the visual evaluation, with MPDL and HPDL generating favorable results. Noise level (where lower noise corresponds to higher visual scores) and liver background homogeneity were found to have a statistically positive correlation (*p* < 0.05), with LPDL generating favorable results. Higher scores when PDL was included suggested improvements in artifacts.

## Discussion

This is the first attempt to evaluate the fundamental characteristics of deep learning-based PDL processing on a digital-BGO PET scanner using both phantom and clinical images. The phantom study was performed to explore the relationship between the β value in BSREM and the PDL models, and to refine the selection of β values in BSREM for to evaluate PDL processing of clinical data, aiming to determine the range of β values that can maximize the physical properties of PDL on Omni Legend. The clinical findings showed that BSREM with PDL significantly improved image quality and enhanced the values for lesions compared with either OSEM or BSREM. The combination of BSREM at β values of 500–600 with MPDL proved particularly effective.

PDL was trained on patient datasets from the Discovery MI PET/CT systems (GE HealthCare), using diverse paired images of non-TOF (input) and TOF (target) BSREM reconstructions collected across multiple clinical centers with varying scanner configurations (3, 4, and 5 rings) [20]. While the training data included only patient images, PDL is designed to predict TOF properties from non-TOF data, allowing its application to phantom studies to replicate TOF effects without additional training. Recent study [32] and the PDL white paper from GE HealthCare [33] have validated the feature of PDL, including its ability to improve image quality and replicate TOF-specific features, through both clinical and phantom experiments, demonstrating its reliability in various imaging scenarios.

The phantom study was used to narrow down the choice of β values applied in the clinical investigation and to optimize the system characteristics [14, 34]. As the β value increased, contrast recovery and background variability decreased, which persisted even when PDL added to BSREM (Fig. 2). The CNR increased as the strength of the PDL models increased, because although noise also increased, the improvement in contrast recovery outweighed it. However, β values that are too high can cause excessive smoothing, which decreases contrast recovery and likely leads to reduced spatial resolution and quantitative accuracy [2, 35, 36]. Thus, careful adjustment of the β value is essential. We clinically evaluated β values of 100, 200, 300, 400, 500, and 600, which resulted in high CNRs in all PDL models. Akamatsu et al. evaluated the performance of 37 images acquired using 23 current PET/CT systems with the same NEMA NU2 phantom and identical settings as in our study [6]. Our CNR results indicated that the Omni Legend outperformed the systems reported in their phantom study in terms of 60- and 90-s/bed images. Moreover, BSREM combined with HPDL on the Omni Legend achieved the highest CNRs among all results reported in that study. The CNR was highest for the Biograph Vision 600 (TOF + PSF) (Siemens) with CNRs of 6.2 and 5.1 for 90 and 60 s, [37] whereas the Omni Legend 32 with BSREM + HPDL achieved CNRs of 6.7 and 5.2 for 90 and 60 s/bed, respectively. These results can be explained by the high sensitivity of the Omni Legend 32 and the SNRs improved by PDL, the effects of which were comparable to TOF [18, 19]. In addition, BSREM + HPDL achieved a CNR with 60-s/bed PET images that was comparable to that of 90 s/bed PET images reconstructed with BSREM (CNR, BSREM + HPDL *vs*. BSREM: 5.2 *vs*. 5.1 for 60 and 90 s/bed, respectively). An under-estimated artifact was observed near the largest spheres with HPDL, likely influenced by edge-related effects introduced by PSF correction, which is automatically incorporated into Q.Clear. PET reconstruction using PSF correction has been reported to cause signal overestimation at object edges and signal underestimation in the surrounding regions, particularly around large spheres [38, 39]. This phenomenon might explain the artifact observed in this study. Lower β values improve spatial resolution [2, 13], leading to a narrower dense edge and a more pronounced undershoot outside the sphere. Since HPDL was trained on PET images with lower β values [20, 33], the under-estimated artifact can become more prominent under HPDL.

Lesion SUV_max_ tended to increase as the strength of PDL intensified from low to high, although the liver SUV_mean_ did not significantly differ among the reconstruction methods. This finding is consistent with a report describing the development of PDL, in which the TOF PET scanner, Discovery MI, was targeted for deep learning, and the BGO non-TOF PET scanner, Discovery IQ, served as the input [20]. During PDL processing, deep learning-based TOF PET networks use a 3D residual U-net as an encoder-decoder and a convolutional neural network (CNN) that was trained in a supervised session. Predicted and target BSREM + TOF PET images were compared using a mean squared error loss function. The β values of the target BSREM + TOF PET images selected for deep learning training averaged 960, 450, and 335 for LPDL, MPDL, and HPDL models, respectively [20]. A lower β value in BSREM improves spatial resolution and enhances quantitation in small lesions such as lung nodules, due to the effect of a relative difference penalty (RDP) as a penalty function [2, 13, 40–42]. The increase in SUV_max_ with HPDL might be explained by the fact that the BSREM + TOF models were trained on lower β values. The β values in digital TOF PET scanners, are usually around 400–500 for BSREM in clinical whole-body ^18^F-FDG PET/CT image reconstruction, which suggests that BSREM + MPDL trained with β = 450 is reasonable [11, 43].

The phantom study revealed both similarities and differences compared to the clinical image assessment [11]. Background variability in the phantom images BSREM + HPDL with β values of 200‒600 was low, but the CNR was higher (Fig. 2). However, BSREM + MPDL with β values of 400‒600 was optimal in terms of noise and SNR in clinical images, whereas both BSREM + MPDL and BSREM + HPDL among all β values provided a better SBR (Fig. 5). Clinical whole-body ^18^F-FDG PET/CT images reconstructed with BSREM + MPDL and β values of 400‒600 offering high SNR, high SBR, and noise equal to or less than that of OSEM + PSF with a 2-mm Gaussian postfilter, would likely be an optimal choice [44].

Precision DL uses deep learning to replicate the effects of TOF [20, 24]. Some studies have shown that deep learning can successfully transform non-TOF PET images into TOF-like images [25, 45]. The time-of-flight improves quantitation, image quality, and lesion detectability on PET images. The present findings suggest that PDL achieves similar benefits. Noise at β values of 400, 200, 300, and 300 for BSREM, LPDL, MPDL, and HPDL, respectively, was equivalent to that of OSEM + PSF but produced significant increases in SUV_max_ (9%, 15%, 18%, and 27%), SBR (16%, 17%, 20%, and 32%), and SNR (17%, 19%, 31%, and 36%), respectively (Table 1 and Fig. 5). In addition, TOF PET improves the localization of annihilation events, which can help mitigate errors in attenuation correction that typically arise from inaccuracies in attenuation maps [46]. The potential of PDL to reduce artifacts caused by respiratory motion is particularly important [47]. We visually confirmed that including PDL reduces these artifacts (Fig. 6). We consider that PDL might mitigate artifacts and further enhance the overall quality of PET images.

Other commercial deep learning-based PET reconstruction techniques, including post-processing methods like SubtlePET (Subtle Medical) [21], AiCE (Canon Medical Systems) [23], and uAI HYPER DLR (United Imaging Healthcare) [22], are trained on paired high- and low-quality PET images, focusing on noise suppression while preserving diagnostic details. In contrast, Precision DL learns the differences between non-TOF and TOF PET images, enabling it to enhance non-TOF datasets beyond noise reduction. We consider Precision DL to offer potential advantages in clinical PET image characteristics, such as lesion detectability, contrast of vertebrae, ribs, lungs, liver, and visceral fat, noise texture, and SUV quantitation [33].

Our visual evaluation found that the best results in terms of noise and liver background homogeneity were delivered by BSREM + LPDL, whereas BSREM + HPDL scored the best for contrast, sharpness, and lesion detectability. In addition, BSREM with MPDL provided the best overall image quality and the highest total mean score. The most effective trade-off was with BSREM + MPDL, which offered lower noise and enhanced lesion detectability, which are critical features for image reconstruction or enhancement. Mehranian et al. also found that BSREM + MPDL balances enhanced lesion detectability with controlled image noise, which contributes to the best diagnostic confidence [20]. Our visual evaluation indicated that overall image quality ranked the highest when images were reconstructed under the conditions of BSREM together with β = 600 and MPDL. This combination might improve image quality, particularly in terms of accurately detecting small lesions. This will increase diagnostic confidence in assessing critical areas such as the liver and mediastinum where background tracer uptake complicates diagnosis [8, 18, 48]. However, additional optimization might be necessary to more precisely elucidate the potential of PDL. Further evaluation under different clinical situations will be important to confirm PDL performance among various imaging applications. Moreover, future studies are needed to assess the feasibility of reducing acquisition time while maintaining or improving image quality with PDL [32] and to evaluate its impact on diagnostic performance by nuclear medicine physician, although these aspects are beyond the scope of this study.

## Conclusions

The combination of BSREM and PDL significantly enhanced the SUV_max_ of lesions and image quality compared with OSEM + PSF. A combination of BSREM, β = 500–600, and MPDL is recommended for oncology body PET/CT imaging when using PDL on the Omni Legend.
